# Human Visual System as a Double-Slit Single Photon Interference Sensor: A Comparison between Modellistic and Biophysical Tests

**DOI:** 10.1371/journal.pone.0147464

**Published:** 2016-01-27

**Authors:** Rita Pizzi, Rui Wang, Danilo Rossetti

**Affiliations:** Department of Computer Science, University of Milan, Milan, Italy; University of Melbourne, AUSTRALIA

## Abstract

This paper describes a computational approach to the theoretical problems involved in the Young's single-photon double-slit experiment, focusing on a simulation of this experiment in the absence of measuring devices. Specifically, the human visual system is used in place of a photomultiplier or similar apparatus. Beginning with the assumption that the human eye perceives light in the presence of very few photons, we measure human eye performance as a sensor in a double-slit one-photon-at-a-time experimental setup. To interpret the results, we implement a simulation algorithm and compare its results with those of human subjects under identical experimental conditions. In order to evaluate the perceptive parameters exactly, which vary depending on the light conditions and on the subject’s sensitivity, we first review the existing literature on the biophysics of the human eye in the presence of a dim light source, and then use the known values of the experimental variables to set the parameters of the computational simulation. The results of the simulation and their comparison with the experiment involving human subjects are reported and discussed. It is found that, while the computer simulation indicates that the human eye has the capacity to detect the corpuscular nature of photons under these conditions, this was not observed in practice. The possible reasons for the difference between theoretical prediction and experimental results are discussed.

## Introduction

According to Richard Feynman, the double-slit experiment contains all the mystery of quantum mechanics [1]. The importance of the theoretical concepts of the double-slit one-photon-at-a-time experiment to the understanding of wave-particle duality and the measurement problem is well known, and extensive literature is available on the subject [2–20]. However, despite the success of quantum theory, quantum mechanics is still open to different interpretations that are typically related to the roles of the observer and the measuring instrument [21]. To deepen our insight into this issue, we intend to use the human visual system as a detector by exploiting the known capacity of the human retina receptors to be activated by a single photon [22–27]; this is an approach recently proposed by other researchers [28–30].

In higher organisms, the eye is a very complex optical system that collects photons from the surrounding environment, regulates their intensity through a diaphragm, focuses the photons through an adjustable assembly of lenses, and conveys them to the retina, which then converts them into electrical signals. These signals are transmitted through a complex pathway that connects the eye to the visual cortex and to other areas of the brain, finally triggering what we call “conscious vision” [31–33]. The retina is a light-sensitive layer at the back of the eye that contains two types of photoreceptors: cones and rods. The eye’s color sensitivity is provided by the cones, which number approximately 6 million, and can be divided into “red” (64%), “green” (32%), and “blue” (2%). Together, they provide light-adapted, or photopic, vision, and are responsible for high-resolution vision [34,35]. The rods are much more numerous than the cones, with potentially 125 million on average in the human eye [36,37]. They are over 1,000 times more sensitive than the cones; however, they are less sensitive to color. The rods are responsible for dark-adapted, or scotopic, vision and, after approximately 30 min of dark adaptation, can reportedly be triggered by single photons under optimal conditions, as will be examined in detail below [38–40].

It has long been confirmed that the eyes of some animal species can sense a single photon [41–44]. However, a single photon cannot trigger a conscious response in the human brain. In the 1940’s, experiments were performed on the sensitivity of the human eye to weak light signals, leading to the conclusion that rod photoreceptors can detect a small number of photons within an integration time of less than 300 ms [25]. Therefore, the human visual system integrates perceived light so that images appear to be stable or moving smoothly. When light is incident on the eye, a minimum number of photons (threshold) must react with the rods within a certain period of time (the “perceptual window”), so that the visual system considers them part of the same stimulus and generates conscious perception. However, estimation of this parameter is complicated by the fact that it depends on the light intensity. Hecht et al. [45,40] conducted a series of studies on the “critical frequency” under various conditions, which can give us some guidance. As we will see in more detail below, we define the critical frequency as the frequency at which the transition from the perception of continuous light to the perception of pulsed light occurs. It is well known that a sufficiently high flickering frequency is perceived as being continuous and, under scotopic conditions, the duration of the perceptive window has been evaluated to be (on average) 100−150 ms. In addition to the already mentioned works, we also consider Chichilnisky’s [46] in vitro studies, which used stimuli of 5−7 photons lasting 10 ms. In those studies, the tested cells could distinguish between two successive stimuli at a temporal separation of 100 ms.

Our knowledge of the responses of the human eye to dim light conditions is based on the classical experiments of Hecht et al. [40]. In their work, these researchers analyzed an earlier study by Langley [47], but re-designed their experiment to re-evaluate the value of the absolute threshold. In [40], the experiments were conducted after the subjects had been left for 30 min in a dark room. The subjects were then asked to indicate if they had seen a flash of light, and the light intensity was then gradually reduced to the minimum perceivable value. As the threshold of vision, Hecht et al. took the light conditions in which the subject perceived a flash of light in 60% of cases. Working with seven subjects, Hecht et al. used a wavelength of 510 μm. For all subjects, the minimum energy necessary for vision ranged between 2.1−5.7×10^−10^ ergs at the cornea, which corresponds to the detection of 54−148 quanta of blue-green light on the cornea. Their corrections for the energy loss due to factors such as corneal reflection, ocular media absorption, and retinal transmission yielded an upper limit of 5−7 quanta required for absorption by the rods, in order to facilitate threshold vision under optimal physiological conditions. They also derived this number from an independent statistical study of the relationship between the intensity of light and the frequency at which it is perceived. The actual number of retinal events varies according to a Poisson distribution, which we will discuss in the Materials and Method section.

However, van der Velden [48] reported a threshold of 2 rather than 5−7. In later work by Barlow in 1956 [49], these discrepancies were explained by the observation that, even when counting single photons, spurious excitation, or retinal noise, is another important factor that affects the absolute threshold value. This explains why the threshold is lowered when the reliability of the responses is reduced and accounts for the disagreement between Hecht et al. and van der Velden. Since this threshold number of photons is distributed on a total area of approximately 350 rods, it was statistically concluded that the rods may respond to a single-photon stimulus [50,51]. However, a serious problem exists in that the parameter on which we can act experimentally is not the number of photons absorbed by the retina, but rather the average number generated externally.

The integrated vision process is an extremely complicated procedure that concerns not only the realm of biology, but also a number of physical processes. First, the photons emitted from a light source should arrive at the retina. During this stage, the majority of photons have a high probability of being reflected by the optical media. Then, the residual photons that finally remain at the retina do not all contribute to triggering of the neural signals. Since the 1950s, the cause of reduced efficiency in light perception has been studied by many authors. For example, Rushton [36,37] estimated that 10% of the light incident on the cornea, or equivalently 20% of the light incident on the retina, is absorbed by rhodopsin in the rods. Some years later, Barlow [52] estimated the overall quantum efficiency, *Q*_*e*_, of the human eye and found that the highest efficiency is almost 5%; this is obtained at near-threshold light intensities. Through research by Baylor, Lamb, and Yau in 1979 [22], who conducted an experiment similar to that performed in [40], along with work by Fuortes and Yeandle concerning intracellular recording in invertebrate photoreceptors [53], the same quantum was confirmed. Taking all these studies into consideration, we can draw a sufficiently rigorous conclusion that the highest overall *Q*_*e*_ of the human eye is approximately 5% in dark-adapted conditions.

This study aims to investigate the feasibility of a Young's single-photon double-slit experiment using the human eye in place of photomultipliers or similar devices. The experimental results are analyzed and interpreted by means of a simulation algorithm. First, the current theory concerning the minimum number of photons required to enable vision in humans and a model of the retinal perception of pulsed light are outlined. Then, the experimental setup used to conduct this experiment and the development of the simulation algorithm are described, and the experimental results are presented. In contrast to the results of the computational simulations, the physical experiments indicate that the human eye, at least in cases of normal visual acuity, cannot be considered equivalent to an artificial measuring instrument.

## Materials and Methods

### Mathematical Preliminaries

#### Temporal summation and Bloch’s law

**Fig 1:**
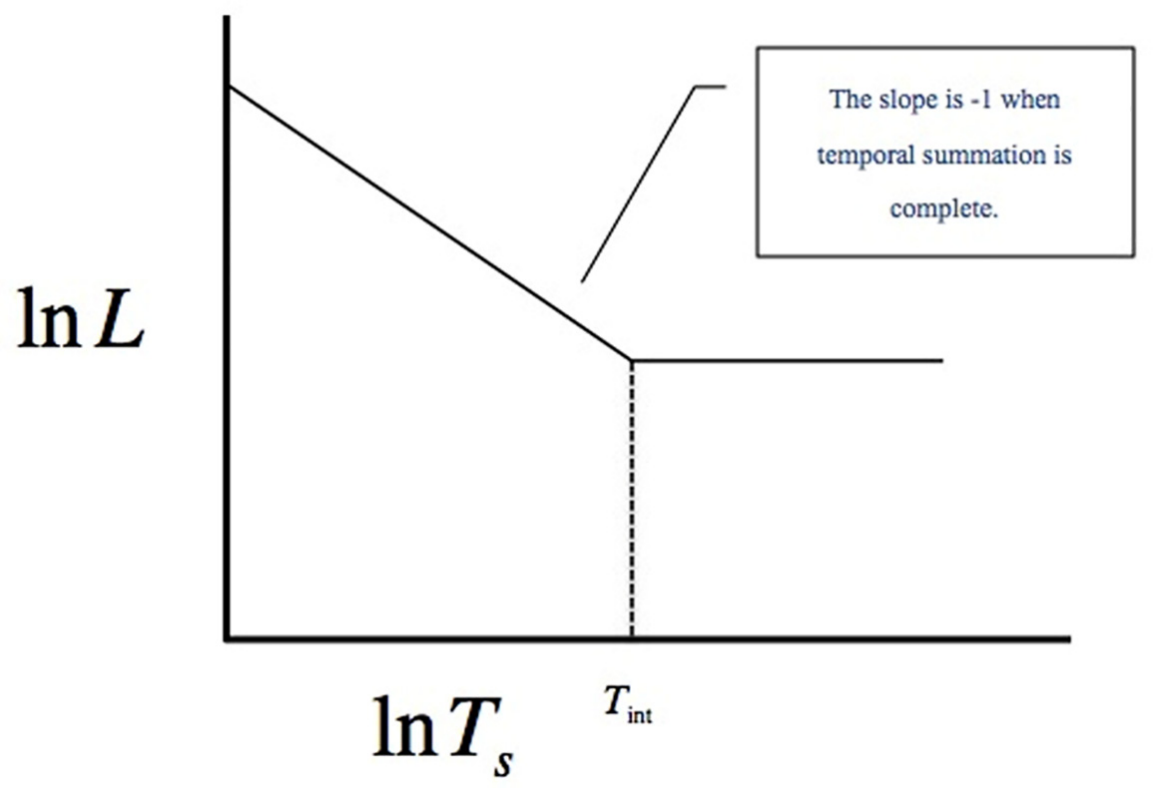
ln *L* vs. ln *T*_*s*_. The relationship between the luminance, *L*, and the duration of the stimuli, *T_s_*, plotted as ln *L* vs. ln *T*_*s*_.

Temporal summation refers to the capability of the human eye to sum up the effects of individual quanta of light over the time domain, within a certain period called the “critical duration” or “critical period.” Bloch’s empirical law [54] affirms that, within this critical duration, the threshold of vision is reached when the total luminous energy is reached. Bloch’s law is expressed as

$$K=LT_{s}^{n},$$ (1)

where *K* is a constant value equated to the total energy required for a conscious perception of light stimuli, *L* is the luminance of the stimulus, *T*_*s*_ is the duration of the stimuli, and *n* measures the completeness of the temporal summation (0 ≤ *n* ≤ 1). No temporal summation occurs when *n* = 0, while complete temporal summation occurs for *n* = 1. By re-expressing Eq 1 in the form

$$L=\frac{K}{T_{s}^{n}}\;,$$ (2)

and taking the logarithm, as shown in Fig 1, we find

$$\text{ln}\;L=\text{ln}\;K-n\;\text{ln}\;T_{s}\;.$$ (3)

If we consider complete temporal summation, which means *n* = 1, we then have

$$LT_{s}=K.$$ (4)

If *L* is sufficiently low, $T_{s}=\frac{K}{L}$ is larger than the integration time, *INT T*_int_, and *K* is not reached within this period of time. Under this condition, the relationship between *L* and *T*_*s*_ can no longer be maintained, and the threshold cannot be reached. This leads to the conclusion that the value of *L* that renders $T_{s}=\frac{K}{L}$ larger than *T*_int_ is too low for the eye to perceive. Hence, we obtain the minimum luminance, $L_{\text{min}}=\frac{K}{T_{\text{int}}}$.

In accordance with Bloch’s law [54], within this critical duration, the threshold is reached when the total luminous energy is reached. Therefore, Bloch’s law states that the total luminous energy for the threshold is a constant value (*K*), and the threshold is reached when *L* and *T*_*s*_ are equal to this constant. The critical duration is shorter for a stimulus of high luminance, as the threshold is reached more quickly. In contrast, this process is slower for a stimulus of low luminance, as a longer period of time is required to sum the quanta and reach the threshold. Temporal summation ceases beyond the temporal *T*_int_. Above this value, the threshold is dependent on the luminance only, rather than the product of both the luminance and duration. In addition, the temporal summation is also affected by other test variables such as the background luminance. It has been shown that temporal integration in the human visual system does not follow Bloch’s law in cases where complex temporal effects are present, as in feature fusion [55]; however, linear energy summation according to Bloch’s law holds for individual elements of a sequence, as in the proposed experiment.

#### Pulsed light perception

In the case of pulsed light, pulses are perceived as separate if the rate at which they are presented is below a certain value. At a certain critical rate, we perceive flickering [56]. Above this threshold, the flickering ceases and we perceive continuous light. This point is called the critical flicker frequency (*CFF*) and is influenced by a number of factors. More formally, the *CFF* is defined as the transition point of an intermittent light source, where the flickering light ceases and appears as a continuous light. Many factors determine our perception of flicker. The Ferry-Porter Law [57,58] states that the *CFF* is proportional to the logarithm of the luminance of the flickering stimulus (*L*). It can be expressed as

CFF=alogL+b, (5)

where *a* and *b* are constants. As the intensity of the stimulus is increased, our perception of flickering also increases. If a stimulus is flickering, decreasing the intensity level eliminates the flicker. In general, the flickering perception ranges from 15−60 Hz, depending on the intensity and wavelength, but some form of flickering can be detected at frequencies of up to almost 100 Hz. For example, a recent paper [59] states that experimental subjects can distinguish images separated by just 13 ms with no interstimulus interval.

**Fig 2:**
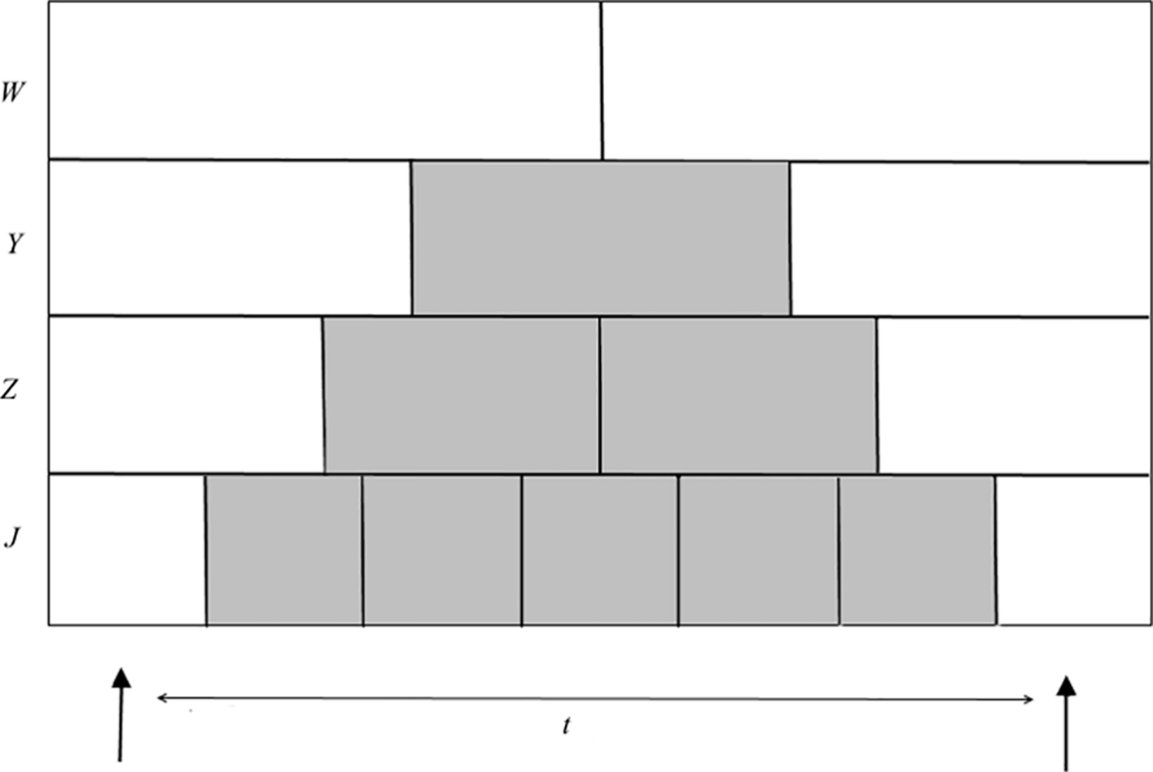
Two stimuli separated by time interval. The speed of integration allows for discrimination between intermittent stimuli. Any cell in each array, *W*, *Y*, Z, and *J*, represents the integration time *T*_int_ in each arrangement. The arrangement of the *Y*, *Z*, and *J* arrays allows for the detection of two different stimuli. The shadows indicate that the eye perceives darkness during these integration times.

However, once the intensity of the source is defined, it is important to understand the empirical laws that rule the ability of the eye to perceive two different stimuli as being separate in time [6,60–67]. In Fig 2, two light stimuli separated by a time interval, *t*, are denoted by two bold black arrows. The detector arrays *W*, *Y*, *Z*, and *J* are divided into cells representing different integration times *T*_int_. For example, in *W*, it is not possible to distinguish between the two flashes. However, in *Y*, *T*_int_ speeds up to 1/3 of the time between the two flashes. The two flashes can then be discriminated successfully since, in the second integration-time cell in *Y*, the eye perceives darkness. In *Z* and *J*, it is also possible to discriminate between the two flashes.

**Fig 3:**
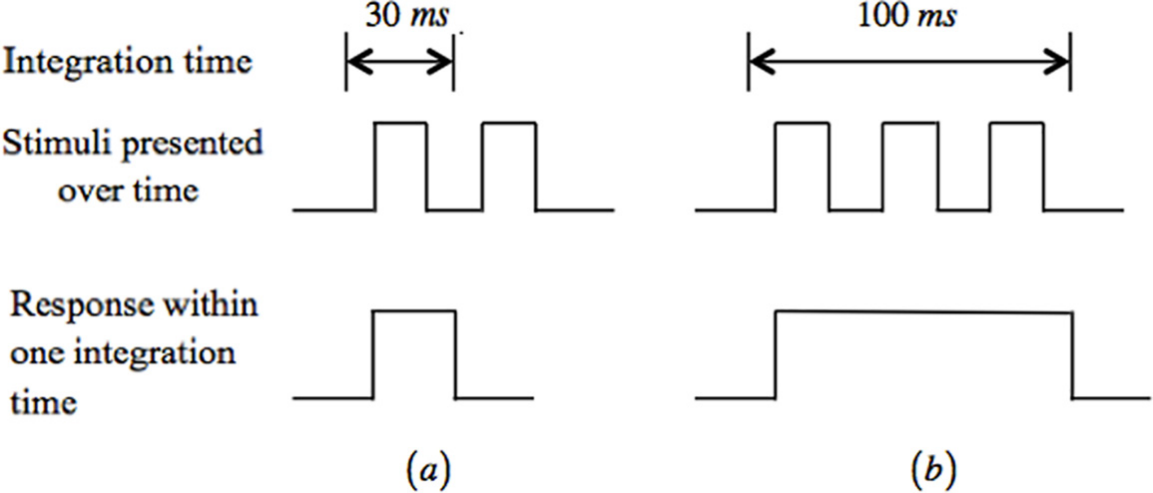
Varying integration times. (a) For a short integration time, flashes can be detected. (b) For a longer integration time, no flashes are perceived. Instead, only one long coherent flash is detected.

Within *W*, the two flashes are regarded as a single coherent flash, whereas in *Y*, *Z*, and *J*, the two flashes are separated. Hence, to detect flashes as being distinct, an appropriate integration time is required (Fig 3). The integration time ranges from approximately 10−15 ms to 0.1 s, depending on the environmental and light-intensity conditions.

#### Light and dark windows

Bloch’s law accounts for the relationship between temporal summation and perception. It is relatively easy to perceive a flash of light in absolute darkness, once it exceeds the vision threshold, but it is very difficult to capture a moment of darkening inside a lasting light beam. In order to consider this relationship between time and perception and to avoid evaluating too many free parameters starting from approximated empirical laws, we condense the free parameters into two empirical conditions: the light window (*L*_*window*_) and dark window (*D*_*window*_). Then, we use experimental values to determine *L*_*window*_ and *D*_*window*_ using the electronic device described below.

**Fig 4:**
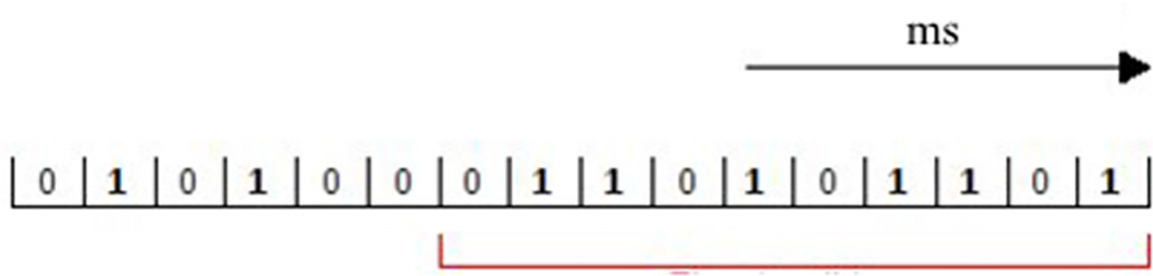
Light window. Each cell represents one millisecond, and the value in the cell represents the number of photons that reach the retina within the same millisecond.

*L*_*window*_ is the maximum time for which a set of photons must be concentrated on the eye to trigger a light perception by the visual system. For example, each cell of the vectors in Fig 4 represents 1 ms, and the value of each cell is the number of photons that have reached the retina photoreceptors in that period of time. Suppose now that the minimum required threshold of photons incident on the rods of the retina, in order to send a pulse to the nervous system, is 6. *L*_*window*_ (highlighted in red) represents the maximum time during which these photons must reach the 6 rods in order for a glow to be perceived and, consequently, until the window (10 ms in length in the example) contains at least 6 photons, the subject continues to perceive darkness.

*D*_*window*_, in contrast, represents the minimum time for which the number of photons remains below the threshold of perception. In fact, if the number of photons remains below the threshold of perception for a period less than *D*_*window*_, the visual system perceives a continuous light beam with no interruption.

#### Flight time

Another important element of the proposed task was to ensure that the photons reached the eye individually. The number of photons simultaneously present inside the instrument was given by *n* = *N*×*t*_*flight*_, where $t_{flight}=\frac{d}{c}$, with the speed of light *c* = 3×10^11^ mm/s and the distance traveled by the photons from the source to the eye, *d* = 890 mm. As $t_{flight}=\frac{890}{3\times 10^{11}}=2.96\times 10^{-9}$ s, it is accurate to state that, inside the tube, the photons reach the eye individually.

### Experimental Setup

This experiment was conducted in the laboratory of Prof. Marco Giammarchi at the Department of Physics of the University of Milan using a suitably modified Teachspin, Inc. (Buffalo, NY) optical bench [68], devoted to the single-photon Young's experiment, along with other measuring devices. The instrumentation was composed of a tube containing a hermetically sealed optical device, 890 mm in length, consisting of a:

- Single-slit collimator;
- Double-slit assembly (double-slit separation: 0.45 mm, slit width: 0.09 mm);
- Detector slit;
- Shutter/ocular.

**Fig 5:**
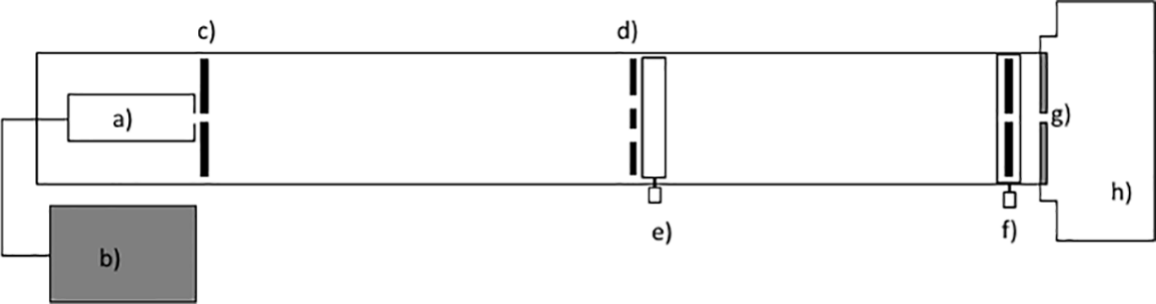
Optical bench. The optical bench consists of a sealed tube containing the following elements: a) light-emitting diode (LED) light source; b) pulsing system; c) optical filter; d) collimator; e) double slit; f) slit blocker with adjustable micrometers; g) detector slit; and h) shutter/ocular. A human eye can be positioned at the ocular, or it can be replaced by a photomultiplier (i). The distance between c) and h) is 890 mm, and the distance between e) and h) is 450 mm.

A detailed description of the optical bench is given in Fig 5.

The widths of the slits and the mutual distances between the source, collimator, double-slit assembly, and the eye were set so as to ensure that the photons reached the photomultiplier or the eye under interference conditions. The light source chosen for the experiment was a NICHIA (Anan, Japan) [69] green light-emitting diode (LED) NSPG520AS-ϕ5 with a wavelength of 520 nm, while the applied optical narrowband filter was an OptoSigma (Santa Ana, CA) [70] with 546.1-nm wavelength and 10-nm bandwidth.

The LED was driven by a homemade circuit providing pulsed or continuous light, which allowed the intensity and frequency of the emission to be adjusted arbitrarily. Although pulsed and continuous mode operation was available, the emission intensity remained unchanged. In the pulsed mode, calibrated voltages were applied, followed by a pause. Both pulses and pauses were generated using a microprocessor. One of the output microprocessor pins controlled a transistor, which was used as a switch to activate/deactivate the LED. Another manual voltage switch facilitated switching between the continuous and pulsed modes. The LED intensity was adjusted using a multiturn steady potentiometer that applied a voltage within the 0−3.2-V range across the LED. Specialized firmware allowed both the duration and the pause to be increased or decreased using separate buttons. The settings were shown on a display connected to the microprocessor. These adjustments could be made in increments of 1 ms, from 0 to 1,000, for both the pulse and the pause.

Before the circuit was used in experimental operations, a digital oscilloscope was employed to ascertain the accuracy of the signals output by the microprocessor, and to determine whether the millisecond values set by the firmware corresponded to the actual durations. As the microprocessor was driven by a controlled quartz oscillator, the obtained accuracy was fully compliant with the experimental requirements. More details on the choice of LED and optical filter, and on the photomultiplier characteristics and measurements, are given in S1 Appendix (Supporting Information).

### Probability Distribution

Since absorption of quanta by the retina corresponds to discrete and independent random events, the number of such events varies according to a Poisson probability distribution [40,71–74]. Suppose that the emission rate of the light source is *r* per unit of time, and that, within the integration time *T*_int_, *rt* is usually sufficiently large. For instance, if the rate of emission is 1000 photons/s and *T*_int_ = 100 ms, 100 photons on average are emitted from the light source for this *T*_int_. Suppose *X* is a random variable with parameter (*k*, *p*), where *k* is the total number of trials. For example, take *k* = 100, as in the above case. In general, *k* = *rt*, where *t* = *T*_int_. We denote *λ* = *kp*. Therefore, the probability of having *n* events occur within a single *T*_int_ is

$$P\left\{X=n\right\}=C_{rt}^{n}p^{n}{\left(1-p\right)}^{rt-n}.$$ (6)

When *rt* is sufficiently large (as in our case), we have

$$P\left\{X=n\right\}=\frac{\lambda^{n}}{n!}e^{-\lambda }\;,$$ (7)

and, thus, the probability of *n* photons striking the retina in each flash obeys the Poisson probability distribution. As *λ* = *kp* = *rtp*, where *p* = 0.5*Q*_*e*_ for each slit (where *Q*_*e*_ is set to 5%, as explained above [66]), the probability of having *n* photons within *t* is

$$P(n)=\frac{{\left(0.5rtQ_{e}\right)}^{n}}{n!}e^{-0.5rtQ_{e}}.$$ (8)

Suppose that perception occurs after *K* photons are detected by the rods. Then, the probability of perception is based on the arbitrary integer *n* being larger than or equal to the threshold value *K*, such that

$$P_{see}=\sum_{n\geq K}\frac{{\left(0.5rtQ_{e}\right)}^{n}}{n!}e^{-0.5rtQ_{e}}.$$ (9)

According to Bloch’s law, this *K* shall be reached during the critical duration. In other words, during the critical duration or, at minimum, *T*_int_, more than *K* photons should be detected by the retinal rods.

As we did not use a laser source, but instead applied a LED source that can be considered to be halfway between a perfect laser source and a thermal light [72,74,75], we considered the possibility that the Poisson probability distribution did not apply to our experimentation, because of the occurrence of photon bunching [76,77]. In S2 Appendix (Supporting Information), this problem is analyzed and overcome.

### Experimental Procedure and Rationale

As mentioned above, the aim of this work is to evaluate the human eye’s performance as a sensor in the context of a “one-photon-at-a-time” double-slit experiment. The rationale behind this approach is as follows. In the case of a typical experiment to investigate the quantum nature of particles, sensors such as scintillators of photomultipliers, together with their processing electronics, are used. In our experiment, we use the human eye as a sensor instead. This work constitutes an example in which the well-known quantum interference is observed by means of the human visual system. On one hand, this introduces the additional complication of the eye's poorly known efficiency in extreme experimental conditions. On the other hand, the work has the conceptual novelty of using a self-conscious system to accomplish the measurement.

In order to gain insight into the process, we implemented a simulation algorithm and compared it with an experiment involving human subjects under the same experimental conditions. Let us consider a double-slit setup under interference conditions. When a photon emitted by the source arrives at the two slits in such a setup, it can be visualized as either a particle or a wave. If we consider it to be a particle, it passes through one of the two slits and proceeds to the eye that observes it at the end of the tube. The eye focuses on both slits and can perceive them clearly. Here, we chose sufficiently separated slits (0.45 mm) that are compatible with the interference conditions of the specific optical bench and light source, but are also easily distinguishable. We were therefore under the same experimental conditions as those of a Young’s experiment setup, where a sensor is used to distinguish the passage of a photon from one slit rather than the other, highlighting its corpuscular nature. Under these experimental conditions, photons should, in fact, be deposited on a screen, resulting in two separate peaks with no evidence of interference. However, as the human eye cannot perceive a single photon, we must apply statistical reasoning. The eye can determine that some quanta are passing through one slit instead of the other if, randomly flowing, they happen to pass through the same slit. They must also be so numerous (i.e., a sufficiently long period of time must be allowed) as to create a perceivable interruption of light at the other slit. The probability of this occurrence depends on many parameters, and is analyzed as below.

In order to evaluate the perceptive parameters exactly, which vary depending on the light conditions and on the subject’s sensitivity, we developed the abovementioned pulsed light system, which allowed us to determine specific parameters for a given subject once the experimental conditions were set. A simulation algorithm was developed and run using a set of experimental parameters, and the obtained results are reported in the next section.

The physical experiment was conducted as follows. First, the subject with the best visual acuity was chosen from a field of several subjects. Then, the light source was set to a continuous flow to determine the lowest perceivable light conditions for the subject. This light intensity was measured using a photomultiplier to ascertain the actual number of photons/ms involved in the experiment and the one-photon-at-a-time condition. Next, the subject underwent an analysis of her visual performance using the ad-hoc pulsed light system mentioned above. In this way, we determined her specific *L*_*window*_ and *D*_*window*_, which are the two parameters necessary to compare her visual performance during the experiment with the simulation results, as explained above. Finally, the subject’s eye observed the illuminated slits for 360 s, during which time the subject identified possible interruptions. The experimental results and their comparison with the simulated data are discussed in the next section.

**Fig 6:**
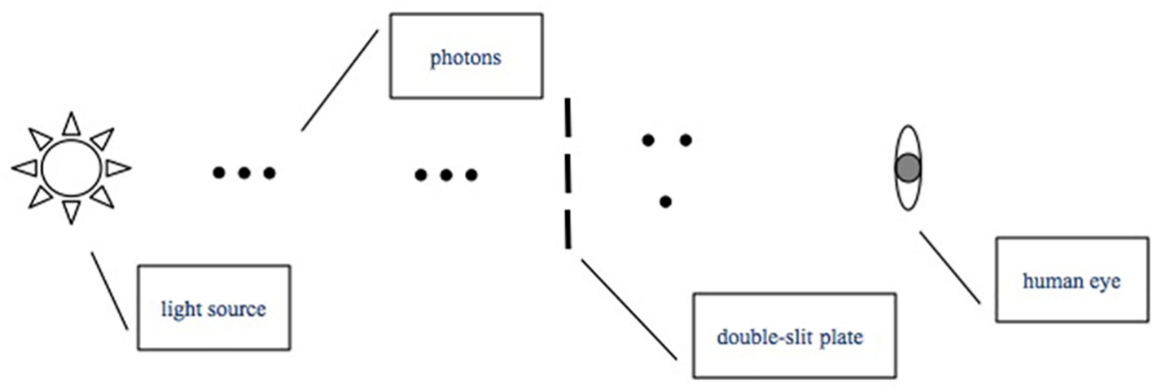
Simplified experimental procedure model. Photons are individually emitted by the source. They pass a double-slit assembly and reach the eye.

The experimental procedure can be understood by consideration of Fig 6. Photons are emitted from a light source and can be treated as being independent of each other. The double-slit plate is positioned in front of the light source, under interference conditions, and the entire setup is sealed inside a long pipe, at the end of which lies an ocular, where the observer puts her/his eye.

After an appropriate time in a dark environment (approximately 30 min, required for the activation of scotopic vision), the subject places her/his eye at the ocular at the end of the tube containing the optical bench. The LED circuit power supply is activated so that the subject can perceive the light.

**Fig 7:**
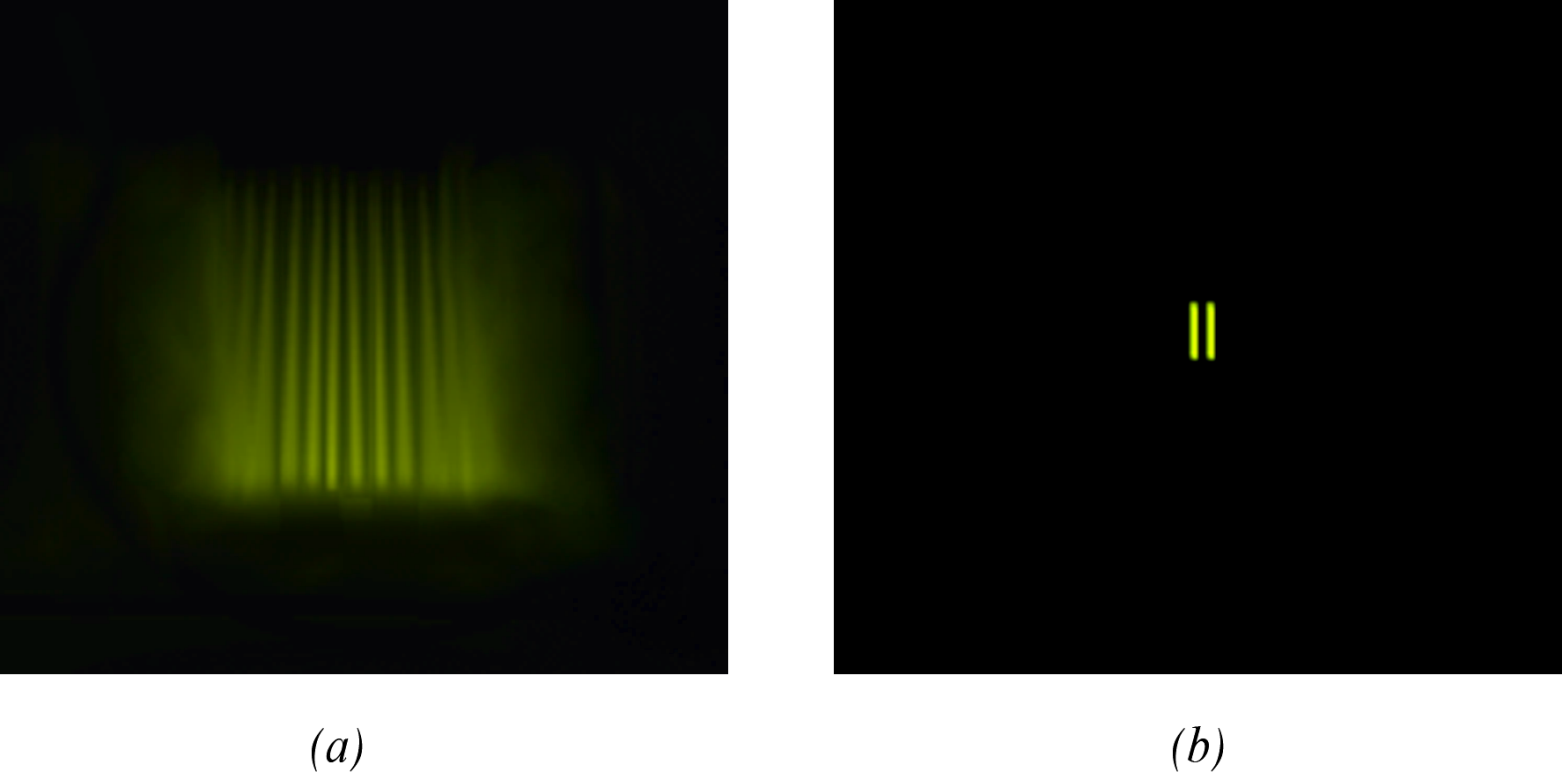
Sketch of observer view. (a) High light intensity. When the photon flux is high, the observer perceives fringes. (b) Low light intensity. For a low number of photons, the observer perceives the two slits. This holds even under interference conditions.

The observer perceives the fringes at maximum LED intensity, as illustrated in Fig 7(A).

Then, the light intensity of the LED is decreased to the minimum perceivable level. As described in detail in the Results section, the image of the interference fringes, which is very clear at the maximum LED intensity, fades as the photon flux decreases. The fringes are perceived as brilliant bars in a region of space that appears located between the eye and the slit position. When the fringes fade, the eye can no longer perceive them. At that stage, there is a clear line of sight between the eye and the illuminated slits and the slits can be perceived separated and bright: the observer perceives the slits as shown in Fig 7(B). In particular, under the specific experimental conditions in which the photons are received one at a time, the eye perceives the two slits in the manner described above. Therefore, all the experimental tests were conducted under the conditions shown in Fig 7(B).

In the course of this experiment, we determined the minimum LED intensity perceivable by the subject with the best visual acuity. We also measured the corresponding number of photons reaching the eye using the photomultiplier, which was found to be approximately 500 photons/s. Then, we determined the *L*_*window*_ and *D*_*window*_ of the subject using the pulsing circuit. The measurement details are given in the next section.

### The Simulation Algorithm

The simulation algorithm modeled the perceptual behavior of the eye during the experiment and included severable tunable parameters:

- *p*: the probability that a single photon passes through either slit (taken as 0.5, according to the random occurrence of independent events following a Poisson distribution);
- alpha: the overall quantum efficiency of the human eye, referred to as *Q*_*e*_ outside of the algorithm and taken as 5%, as explained above;
- threshold: the absolute threshold of light perception, i.e., the minimum number of photons required to perceive light, taken as 5−7, as explained above;
- *L*_*window*_: the time under conditions of darkness required for the eye to be able toperceive incident light;
- *D*_*window*_: the time that the eye requires to perceive a moment of darkness once the light beam is interrupted;
- *N*: the photon emission rate from the light source in one unit of time. For instance, if the photon emission rate is 1,000 photons/s and 1 ms is taken as one unit of time, *N* is 1 photon/ms;
- *L*_*period*_: the duration of one continuous photon emission in a pulsed mode. For instance, if the light source emits for 30 ms and is then paused for 40 ms, *L*_*period*_ is 30 ms;
- *D*_*period*_: the pause in the emission. Using the same example as above, *D*_*period*_ is 40 ms;
- totaltime: the total time of a single experiment, expressed in ms.

The original version of the algorithm was first written using a JAVA platform (Oracle America, Inc., Redwood City, CA) [20] and was then further optimized and implemented in *MATLAB* 6.1 (Release 12.1; MathWorks, Inc., Natick, MS).

**Fig 8:**
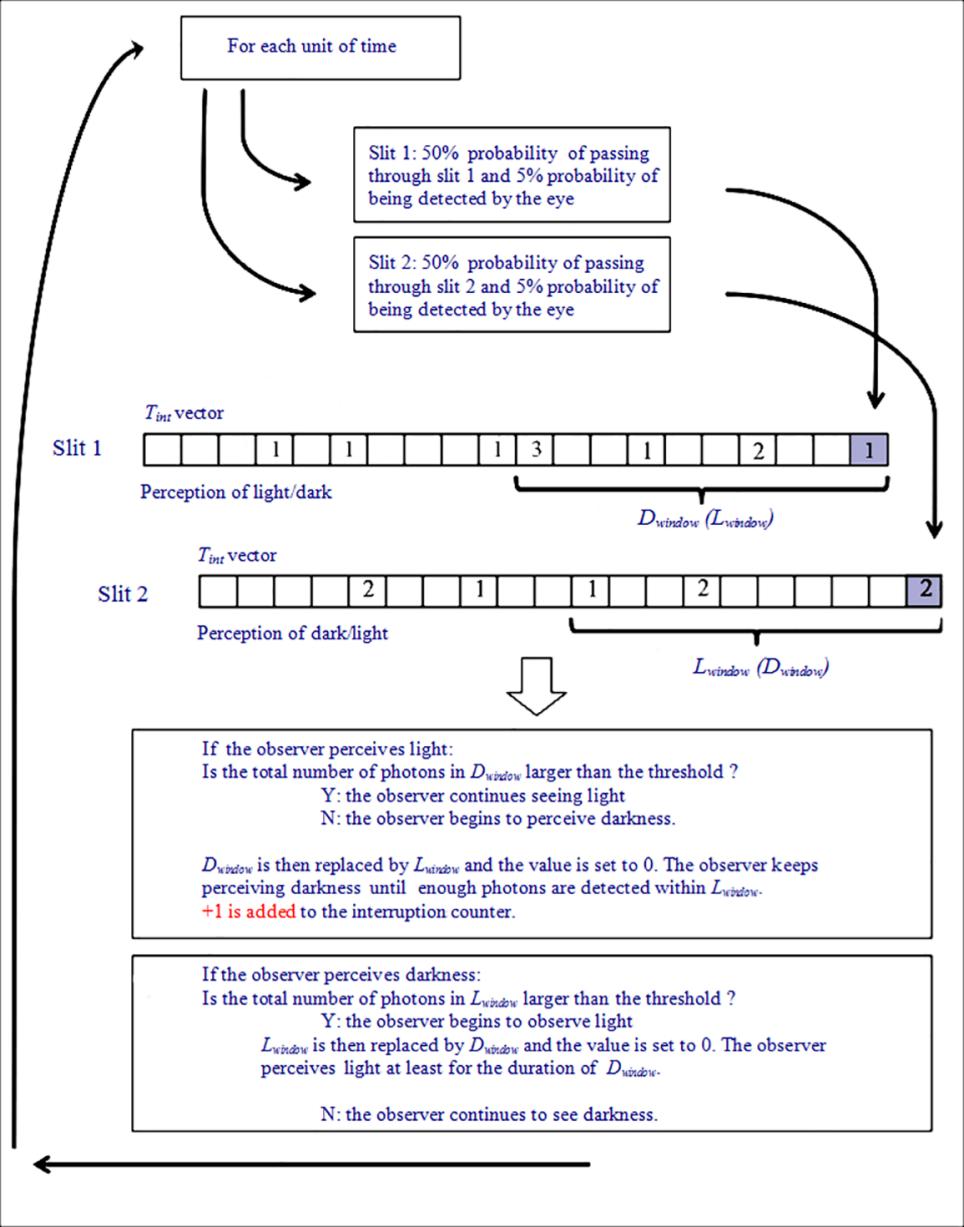
Process diagram of simulation algorithm and rationale. A time vector models the integration time *T*_int_ related to the observation of each slit. Each cell in the vector represents one unit of time. If, within a certain unit of time, *i* photons are detected by the retinal rods, we write *i* in the cell that corresponds to that unit of time. The interruption counter is increased depending on the total number of photons present in the observer’s *D*_*window*_ and *L*_*window*_.

The entire procedure is outlined visually in Fig 8. First, we created a time vector as a model of *T*_int_. In this setup, the vector length depends on the window it represents, as each cell in the vector represents one unit of time. For example, if the time vector is for *D*_*window*_, with a value of 100 ms, this vector is divided into 100 individual cells, each of which represents 1 ms (taking 1 ms as a time unit). Therefore, if *i* photons are detected by the retinal rods in the *n*th ms, we write the number *i* in the *n*th cell. Then, we calculate the sum of these numbers inside this time vector, which indicates the total number of photons detected by the retinal rods within this time window. If the result is larger than the threshold of vision, the eye perceives light at this moment. Conversely, if the result is lower than the threshold, the eye perceives darkness. Hence, we can calculate the total number of photons from the *(n-Dw+1)*st unit of time to the *n*th unit of time, where *Dw* is the length of *D*_*window*_ (in the case of *L*_*window*_, *Dw* should be replaced by *Lw*). If the sum of this calculation is greater (less) than the threshold we selected in the first place, then, at the *n*th unit of time, the eye in our simulation perceives light (darkness).

## Results and Discussion

### Simulation Results

The simulation was conducted for several free parameter values, taking into account the fact that they can vary significantly in response to a number of environmental and eye conditions, as we have seen previously. Tables 1 and 2 show the number of interruptions/5,000 ms for a *Q*_*e*_ of 0.05. The yellow cell indicates the *L*_*window*_ and *D*_*window*_ of the experimental subject. The experiment was conducted on three independent subjects and the final measurements were taken from the subject with the best visual acuity.

**Table 1:** Simulation results for a 5,000-ms period at 500 photons/s and a threshold of 6. The average values over 6 trials are given. L indicates *L*_*window*_, D indicates *D*_*window*_.

		L	L	L	L	L	L	L	L	L
		20	30	40	*65*	100	150	200	250	300
**D**	**300**	3	5	5	9	12	14	18	21	19
**D**	**250**	3	3	7	9	19	20	20	21	19
**D**	**200**	5	5	5	9	12	22	27	30	27
**D**	**150**	3	5	6	11	17	30	35	33	33
**D**	**100**	3	5	5	7	20	25	32	40	41
**D**	**50**	5	3	5	8	13	44	42	40	47
**D**	***20***	5	5	7	***7***	11	31	52	51	52

The table shows the number of interruptions calculated via simulation, where the possible *L*_*window*_ and *D*_*window*_ of the observer were varied. The number of interruptions predicted for a subject with the same visual acuity as our best-performing subject during the test (65 for *L*_*window*_ and 20 for *D*_*window*_) is shown in bold and Italic (= ***7***). This value refers to the average number of interruptions from slit 1, from slit 2, and from both slits. The other cells indicate that the average number of interruptions decreases with decreased *L*_*window*_, and remains almost steady with decreased *D*_*window*_ (at fixed *L*_*window*_). A zero-interruption result is never obtained for *L*_*window*_ and *D*_*window*_ values compatible with normal human-eye visual acuity.

**Table 2:** Simulation results for a 5,000-ms period at 500 photons/s and a threshold of 8. The average values over 6 trials are given. L indicates *L*_*window*_, D indicates *D*_*window*_.

		L	L	L	L	L	L	L	L	L
		20	30	40	*65*	100	150	200	250	300
**D**	**300**	<1/1000000 s	~1/100000 s	~1/50000 s	6	9	16	16	25	21
**D**	**250**	<1/1000000 s	~1/100000 s	~1/50000 s	5	7	15	17	22	24
**D**	**200**	<1/1000000 s	~1/100000 s	~1/50000 s	5	5	9	19	21	21
**D**	**150**	<1/1000000 s	~1/100000 s	~1/50000 s	5	11	18	26	35	27
**D**	**100**	<1/1000000 s	~1/100000 s	~1/50000 s	5	7	21	18	30	33
**D**	**50**	<1/1000000 s	~1/100000 s	~1/50000 s	7	11	22	31	35	42
**D**	***20***	<1/1000000 s	~1/100000 s	~1/50000 s	***5***	11	17	37	38	46

The table shows the number of interruptions calculated via simulation, where the possible *L*_*window*_ and *D*_*window*_ of the observer were varied. The number of interruptions predicted for a subject with the same visual acuity as our best-performing subject during the test (65 for *L*_*window*_ and 20 for *D*_*window*_) is shown in bold and Italic (= ***5***). This value refers to the average number of interruptions from slit 1, from slit 2, and from both slits. The other cells indicate that the average number of interruptions decreases with decreased *L*_*window*_, and remains almost steady with decreased *D*_*window*_ (at fixed *L*_*window*_). A zero-interruption result is never obtained for *L*_*window*_ and *D*_*window*_ values compatible with normal human-eye visual acuity.

**Fig 9:**
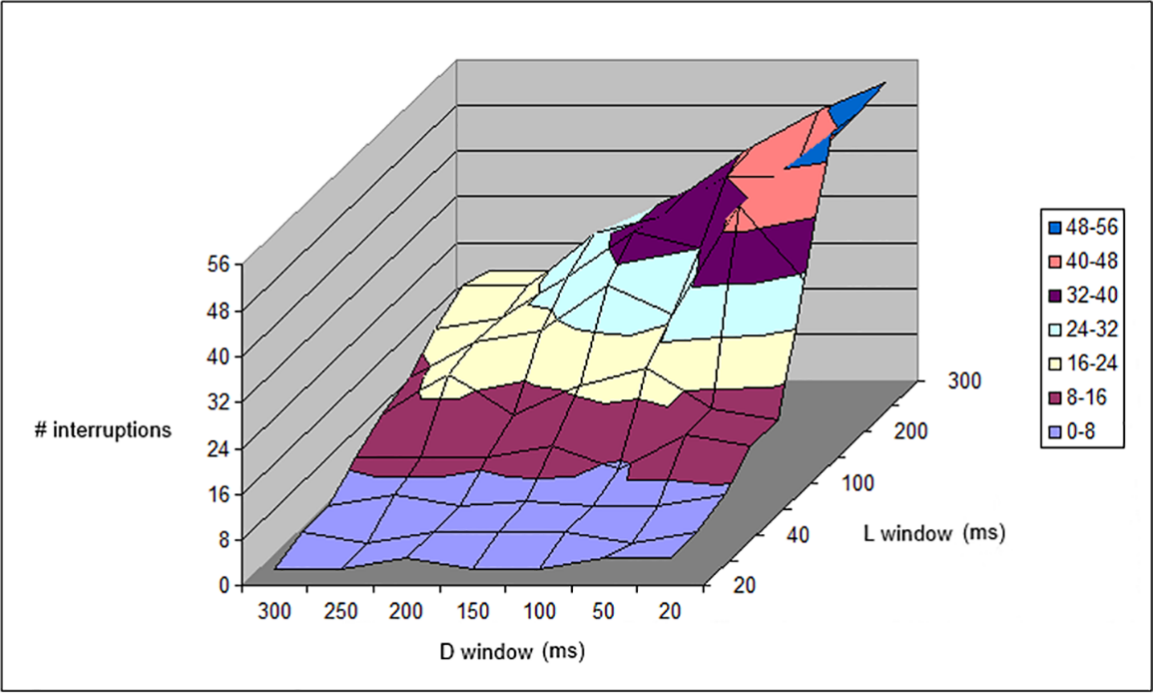
Plot of data given in Table 1. Number of interruptions with respect to *L*_*window*_ and *D*_*window*_ length, for a 5,000-ms period at 500 photons/s and a threshold of 6, as reported in Table 1.

**Fig 10:**
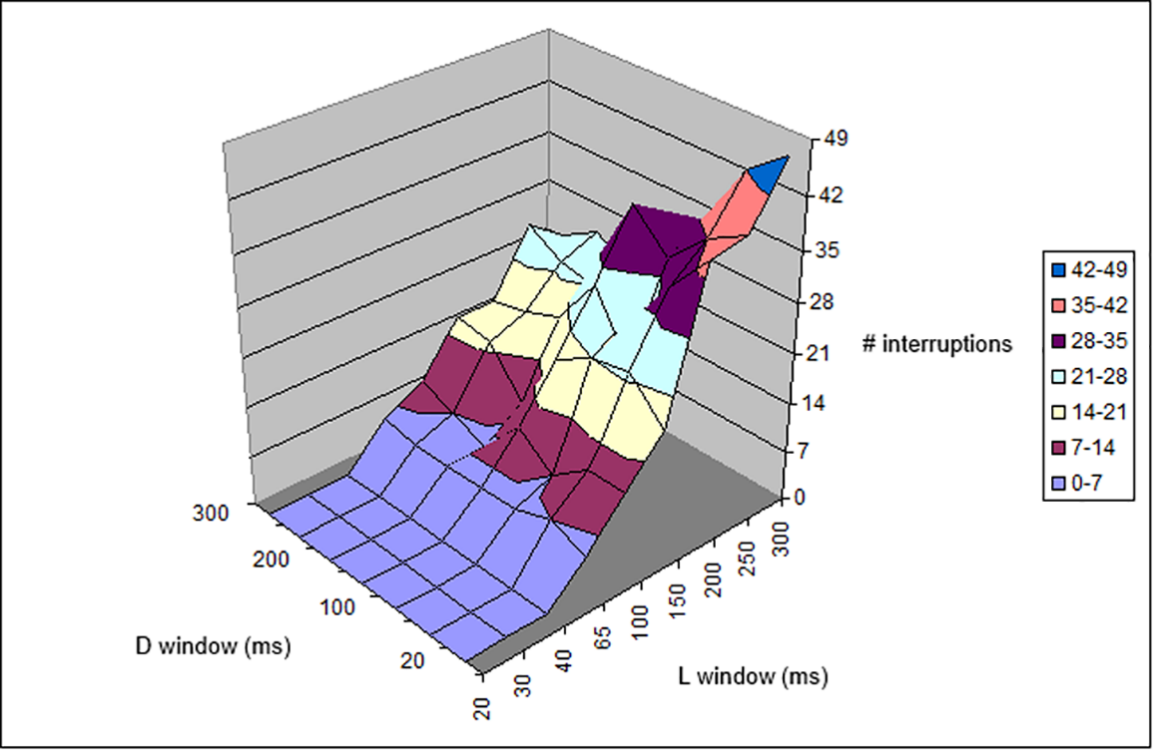
Plot of data given in Table 2. Number of interruptions with respect to *L*_*window*_ and *D*_*window*_ lengths, for a 5,000-ms period at 500 photons/s and a threshold of 8, as reported in Table 2.

The perceived interruptions of the light stream forecast by the simulation algorithm were recorded for an *L*_*window*_ of up to 20 ms, with 500 photons/s corresponding to the passage of 10 photons, a number very near to the minimum threshold (6 (Table 1, Fig 9) or 8 (Table 2, Fig 10) photons) necessary to perceive light. This *L*_*window*_ is simply a theoretical characteristic of a “perfect” eye, which does not require a longer *T*_int_ to detect a flash of light. As detailed in the previous section, the different combinations of dark and light sensitivity show interruptions that could also be due to the eye’s difficulty in following the random photon succession. It can be seen in the tables that, although the number of interruptions increases slightly with a decrease in *D*_*window*_ for constant *L*_*window*_ (as the visual acuity increases), the number of interruptions tends to decrease when the visual acuity increases (low *L*_*window*_ and *D*_*window*_). This indicates that many of the interruptions in the case of high *D*_*window*_ and *L*_*window*_ are due to poor visual acuity (high integration time). However, at low *L*_*window*_ and *D*_*window*_, the number of interruptions reaches a plateau, which indicates a lack of dependence on the windows; thus, these interruptions are “real”, i.e., they are due to a real lack of photons rather than the visual deficit caused by the sequence of *L*_*window*_ and *D*_*window*_.

A subject with 65/20 windows should perceive approximately 1 interruption/s, and the number of interruptions for high *L*_*window*_ should be perceived as flickering. At a higher threshold, the statistics prevent even a “perfect” eye from perceiving interruptions during a short observation time, even though, in principle, a long observation could allow an interruption to be detected. In general, if for the same visual performance, more (8) photons are required for perception, this eye would typically fail to detect some interruptions, in comparison with a subject with superior-acuity eyesight (6 photons). However, in the case of a long *L*_*window*_, this eye would also perceive some false interruptions, due to the simultaneous need for more photons and for a long, continuous light emission.

**Fig 11:**
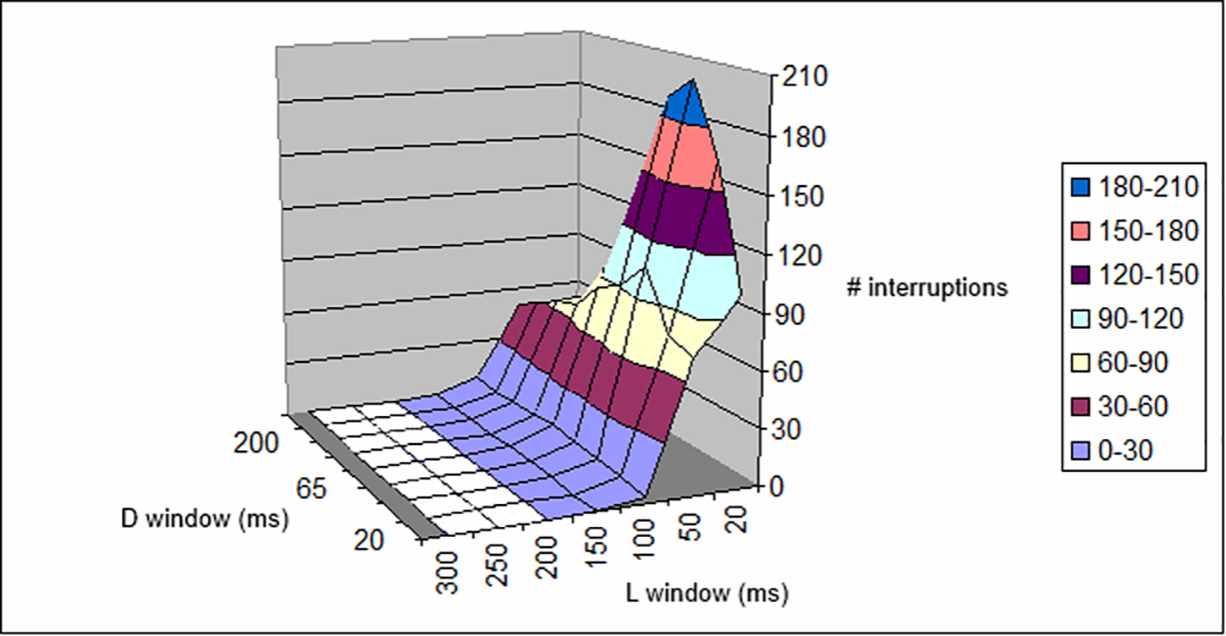
Plot of data given in Table 3. Number of interruptions with respect to *L*_*window*_ and *D*_*window*_ length, for a 5,000-ms period at 5,000 photons/s and a threshold of 6, as reported in Table 3.

**Fig 12:**
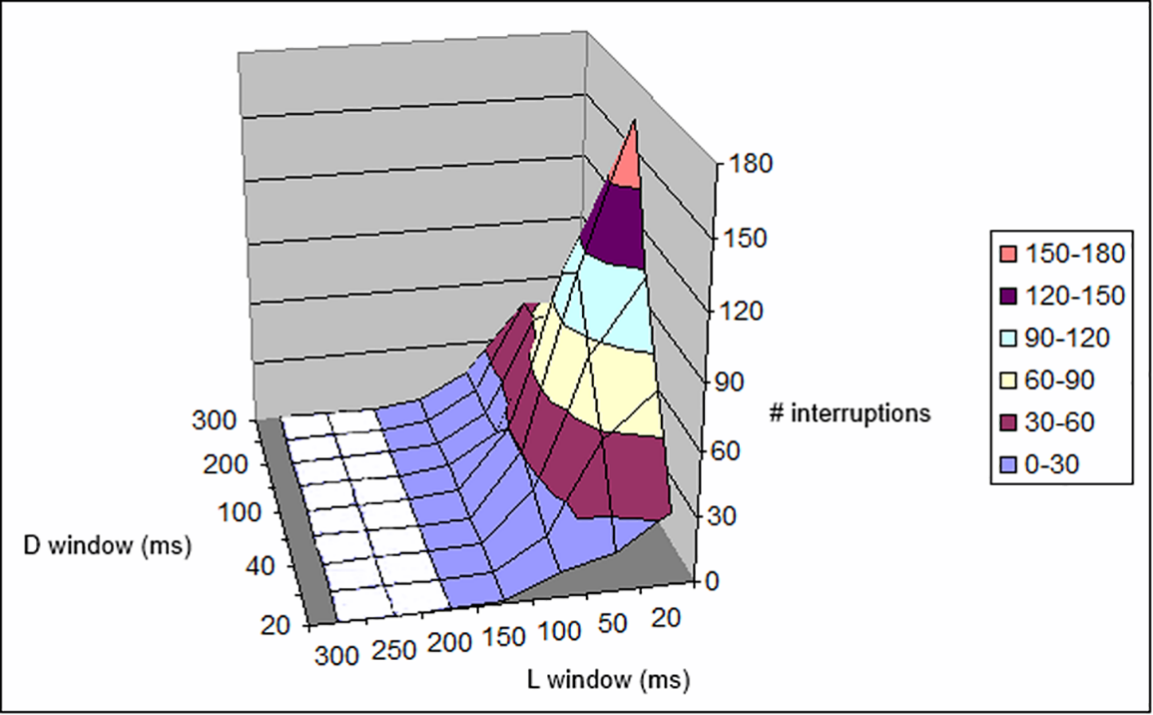
Plot of data given in Table 4. Number of interruptions with respect to *L*_*window*_ and *D*_*window*_ length, for a 5,000-ms period at 5,000 photons/s and a threshold of 8, as reported in Table 4.

The tables confirm that the choice of 500 photon/s in our experimental setup is correct, i.e., this is the appropriate value to obtain a consistent number of observations within a reasonable observation time. In fact, if the number of photons/s is increased (5,000/s; Table 3, Fig 11 and Table 4, Fig 12), the interruptions occur too swiftly and, for high *L*_*window*_ and *D*_*window*_, the eye is physiologically unable to distinguish between them. By increasing the visual acuity, the low numbers of interruptions indicated by the simulations mean that it is impossible for the eye to perceive them all. A “perfect” eye should, in principle, perceive all the interruptions (it could perceive 1,000 ms/(20 + 20) ms = 25), most likely in the form of flickering. It can be seen that almost the same observations hold at 5,000 photon/s and at a threshold of 8 photons.

**Table 3:** Simulation results for a 5,000-ms period at 5,000 photons/s and a threshold of 6. The average values over 6 trials are given. L indicates *L*_*window*_, D indicates *D*_*window*_.

		L	L	L	L	L	L	L	L	L
		20	30	40	*65*	100	150	200	250	300
**D**	**300**	0	0	0	0	0	0	0	0	0
**D**	**250**	0	0	0	0	0	0	0	0	0
**D**	**200**	0	0	0	0	0	0	0	0	0
**D**	**150**	0	0	1	1	1	0	1	0	1
**D**	**100**	3	6	7	8	10	8	8	6	8
**D**	**50**	72	77	107	91	84	68	64	57	50
**D**	***20***	101	164	202	***190***	121	87	66	55	45

The table shows the number of interruptions calculated via simulation, where the possible *L*_*window*_ and *D*_*window*_ of the observer were varied. The number of interruptions predicted for a subject with the same visual acuity as our best-performing subject during the test (65 for *L*_*window*_ and 20 for *D*_*window*_) is shown in bold and Italic (= ***190***). This value refers to the average number of interruptions from slit 1, from slit 2, and from both slits. The other cells indicate that, with a high number of photons, the eye can still perceive interruptions, except in the case of high *D*_*window*_. Many interruptions are visible in the case of normal human-eye visual acuity. In the case of a visual deficit (large *L*_*window*_ and *D*_*window*_), the subject cannot distinguish any interruptions.

**Table 4:** Simulation results for a 5,000-ms period at 5,000 photons/s and a threshold of 8. The average values over 6 trials are given. L indicates *L*_*window*_, D indicates *D*_*window*_.

		L	L	L	L	L	L	L	L	L
		20	30	40	*65*	100	150	200	250	300
**D**	**300**	0	0	0	0	0	0	0	0	0
**D**	**250**	0	0	0	0	0	0	0	0	0
**D**	**200**	0	0	0	0	0	0	0	0	0
**D**	**150**	1	1	1	1	2	3	2	3	3
**D**	**100**	11	15	24	31	30	17	15	16	15
**D**	**50**	17	57	84	111	102	72	61	58	42
**D**	***20***	33	77	117	***177***	147	87	66	54	45

The table shows the number of interruptions calculated via simulation, where the possible *L*_*window*_ and *D*_*window*_ of the observer are varied. The number of interruptions predicted for a subject with the same visual acuity as our best-performing subject during the test (65 for *L*_*window*_ and 20 for *D*_*window*_) is shown in bold and Italic (= ***177***). This value refers to the average number of interruptions from slit 1, from slit 2, and from both slits. The other cells indicate that, with a high number of photons, the eye can still perceive interruptions, except in the case of high *D*_*window*_. Many interruptions are visible in the case of normal human-eye visual acuity. In the case of a visual deficit (large *L*_*window*_ and *D*_*window*_), the subject cannot distinguish any interruptions.

The minimum number of interruptions for the subject with the best visual acuity, best visual performance (*L*_*window*_/*D*_*window*_ = 65/20), and an average vision threshold of 7 photons was used to evaluate the observation time needed to ensure an interruption with 99% probability. The resultant time was 3,083.777 ms (the right extreme of the confidence interval), and an observation time of 360,000 ms was adopted on a controlled basis.

### Experimental Results

The preliminary measurement necessary to conduct the experiment was the evaluation of the minimum perceivable LED intensity. This was determined as being 61.8 μA for the subject with the best visual acuity. At this light intensity, the quantity of photons reaching the eye was evaluated using the photomultiplier and digital counter, with a dark noise of 7.5 shots/s and a voltage of 760 V. The results were as follows:

- No. photons/s: 17.73;
- No. of photons/s taking a *Q*_*e*_ of 4% (as stated by the photomultiplier manufacturer): 443.33;
- 99% confidence interval: [387.32, 499.33].

Thus, to obtain 99% certainty concerning the validity of the computations resulting from the simulation procedure, a photon/s value of 500 was considered (as mentioned above).

The second preliminary measure was the evaluation of the *L*_*window*_ and *D*_*window*_ of the subject using the above-described pulsing circuit. The pulsing circuit was initially activated with sufficiently large pause and pulse durations to render the two slits easily observable. Gradually, the pulse and pause lengths were decreased, until a minimum necessary light intensity/pulse duration/pause duration configuration was reached that allowed the subject to perceive an interruption between two pulses. These values were fixed as the *L*_*window*_ and *D*_*window*_ of the subject.

The observation phase was aimed at highlighting the possible perception of interruptions of the light flow from one or both slits. As stated previously, the guiding aim of our project was to verify that the number of photons required for the human eye to perceive light could be sufficiently low to allow for the perception of a random interruption of the photon stream within a relatively short period of time. The statistical procedure confirmed this view. In fact, based on the probability distribution of the specific flow of the photons, a non-zero probability exists that the photons pass by only one of the two slits in sufficient numbers to cause an interruption of light perception in the other slit.

Following the simulation, the physical experiment was conducted as previously outlined. Here, the direct observation of the two slits by the subject continued for 360 s, well above the period allowed for the software simulation for the appearance of light interruption. However, no light interruptions were perceived. The observation was conducted with a subject certified with optimal visual acuity (10/10 eyesight with no visual defects). On the basis of Table 1, which is the most adherent to the real experimental conditions following the reported literature on the threshold of vision, the subject should have perceived not less than 500 interruptions during this observation. Further test observations under identical or very similar experimental conditions to those of this and the other subjects (who exhibited similar visual parameters) continued informally for a total of >3 h with no detection of interruption or flickering.

With regards to data acquired from human subjects, a number of points must be made. First, it must be emphasized that interruption due to eye blinking is of course below the threshold of visibility of the interruption itself [78]; thus, the duration of the eye blink is below the *D*_*window*_ value. This implies that blinking does not influence the experimental results as regards the number of interruptions perceived by the eye. The possibility that the observer could miss many interruptions due to inattention, fatigue, or transient visual deficits must be taken into consideration. However, the principal subject did not perceive any interruptions or flickering of any kind, indicating a visual experience that differed completely from the predicted response. The other three subjects reported slightly different *L*_*window*_ and *D*_*window*_ values, but none perceived interruptions over the >3-h total observation time, for a large number of experimental sessions. Based on the simulation results, the overall missed interruptions should have numbered in the thousands, and this number does not seem to correspond with the occasional occurrence of a transient visual deficit. On the other hand, the presence of a transient visual deficit could be a factor in the perception of spurious interruptions. However, none of the subjects experienced interruptions of any kind during the >3-h observation time; therefore, no investigation was conducted into transient visual deficits as the origins of perceived interruptions.

Besides the results related to the specific two-slit problem, the experiment allowed us to make additional interesting observations about the biophysics of the eye under different lighting conditions. In particular, we verified the temporal resolution threshold of the human eye under the simultaneous variation of several parameters. As indicated by the data shown in Tables 1–4, this value is not fixed for any subject, and has a complex relationship with the number of photons and the presence of a suitable dark interval between the light flashes. In other words, we verified that the values of *D*_*window*_, *L*_*window*_, and the number of photons are strictly correlated for each subject. Besides, each subject appears to possess not one, but rather a set of valid parameter configurations. For example, setting a dark interruption longer than the subject’s *D*_*window*_ allows a shorter *L*_*window*_ to be obtained. In contrast, *L*_*window*_ can be decreased only if the interruption that precedes and succeeds the light pulse is sufficiently long.

Another interesting aspect of this study was the possibility of exploring the subjects’ direct visual experiences of the interference fringes formed using a double-slit apparatus with different light conditions. We found that the observer clearly perceives the interference fringes under bright-light conditions, which capture the eye’s focus. As the photon flux decreases, the interference fringes disperse, becoming extremely diluted and imperceptible; this causes the eye to focus on the slits directly, which are perceived as being farther than the fringes. Further, the slits appear to be clearly illuminated, even when the photons are transmitted individually. The described experiments were conducted under these light and perception conditions, which are illustrated in Fig 7B.

It must be noted that the perception of the interference fringes also appears to be dependent on other parameters, particularly the vertical viewing angle above the horizontal. As a result of the spatial symmetry of the experimental setup, inclination of the point of view by a small number of degrees directs the sight through a portion of space with lower interference-wave intensity. This induces the eye to focus on the slits instead of the fringes, even under conditions of high light intensity.

## Conclusions

The scientific contribution made by the computational and experimental findings presented in this work constitutes an up-to-date and precise evaluation of the performance of the human eye under extremely dim lighting conditions, and of the minimum number of photons perceivable by the eye for pulsed and continuous light. In fact, the literature on this topic that has been cited above [23–27, 31–66] incorporates a large number of related experiments dating back over several decades, performed using dated methods or equipment. On the other hand, more recent works cover other aspects of the problem considered here and draw different sets of conclusions.

The introduction of the novel concept of light and dark windows and the computational modeling approach employed in this paper facilitated examination of the behavior of the human eye under different light conditions, and under the simultaneous variation of several parameters.

The use of a quartzed pulsing system allowed the timing of the eye's reaction to light to be finely tuned, and facilitated the precise recording of data. In future, dedicated experiments using these novel parameters and the specified experimental setup may shed light on this interesting aspect of the eye's biophysics as, at present, sparse experimental data exists. Further, the computational simulation allowed the interaction process between photons and the eye to be rationalized and modeled. This approach may constitute a valuable future tool for exploration of further aspects of this specific problem and the biophysics of the eye in general.

Moreover, this study made it possible for subjects to report on their experience of direct perception of the interference fringes formed by a double-slit apparatus under different light conditions. The interference fringes were found to be easily visible, but this did not exclude the possibility of perceiving the illuminated slits, which depends both on the number of photons and on the observer’s point of view.

As regards the evaluation of the human eye as a single-photon sensor without artificial measuring instruments, this research highlighted a divergence between the computational simulation, developed based on biophysical parameters, and the experimental results obtained using the human eye as a sensor. In fact, the computational simulation indicated that the visual performance of the eye is adequate for detection of a photon’s passage through a two-slit apparatus, highlighting the corpuscular nature of these objects. However, the experimental results obtained using the human eye as a sensor did not confirm the hypothesis that the eye could be considered equivalent to a measuring instrument. The experimental results were found to diverge significantly from the predicted performance, in that no interruptions were detected during a 360-s observation period, although detection of at least 500 interruptions was predicted. It is difficult to attribute this significant discrepancy between experiment and theory to experimental errors, which should have a less significant effect.

Nonetheless, experimental errors should be taken into account, because of the technical difficulty of the experiment. In particular, this experiment should be repeated under less restrictive experimental conditions, without adopting the most extreme visual threshold for the subject, so as to facilitate detection of interruptions. In fact, Tables 1–4 show that, under a wide range of values of *D*_*window*_, *L*_*window*_, and the number of photons, the number of interruptions is sufficiently low that the interruptions are clearly distinguishable, but sufficiently high that the risk of experimental errors due to proximity to the extremes of the visual parameters is minimized. Such experimental errors include errors in the evaluation of the biophysical parameters and a lack of interruption detection due to inattention, fatigue, or transient visual deficits.

Thus, it is apparent that this experiment should be replicated in other laboratories before theoretical conclusions are drawn, in order to confirm these findings. The values of the theoretical biophysical parameters and the simulation procedure could also be tuned further. Many other subjects with different visual acuities should also be recruited and tested.

After this necessary stage, and with extreme caution, the findings may be discussed in the context of the most influential theories on the foundations of quantum mechanics [79–138]. Should further experimentations confirm the present results, they could lead to reflections on possible differences between quantum measurement accomplished by direct human observation and by means of artificial devices. On the other hand, if a different result should emerge from the future experiments, showing that the human eye can indeed detect the corpuscular nature of photons, this experimental model could be used to confirm the equivalence between artificial measurement devices and human eyes as detectors, or causes, of quantum wave collapse.

The issue of quantum measurement, considering the role of the observer and the position of the Heisenberg/von Neumann cut [79–102], has been debated in the past by the majority of the founding fathers of quantum mechanics, and it remains a topic that incites passionate discussion. However, this debate is far from resolution [103–139], recently involving also quantum processes in vision [140–151]. Therefore, the most common approach as regards quantum mechanics interpretations adopts the useful concept known as "For All Practical Purposes" (FAPP) [152], which allows physicists and physics to progress unabated and effectively, while considering the answer to this question as being beyond the current reach of theoretical physics.

## Supporting Information

S1 Appendix Discussion of the choice of LED and optical filter, and of the photomultiplier characterstics and measurements. (DOC)

S2 Appendix Discussion of the bunching problem. (DOC)

S1 Fig Classic slow-phase adaptation curves. After approximately 20-min exposure to dark conditions, the rods achieve maximum sensitivity.
(TIF)

S2 Fig Maximum sensitivity for scotopic vision (from [33], p. 168). Overlay of measured data (solid line with experimental values) with theoretical prediction (dotted line).
(TIF)
