# Supplementary material for: Human Visual System as a Double-Slit Single Photon Interference Sensor: A Comparison between Modellistic and Biophysical Tests
Source: PLoS One. 2016 Jan 27;11(1):e0147464. doi: 10.1371/journal.pone.0147464 (PMC4729532; doi:10.1371/journal.pone.0147464)
Supplement: S1 Appendix — (DOC) [file pone.0147464.s001.doc]

#### S1 Appendix

#### Choice of LED and Optical Filter

The choice of the LED source was based on the need to limit the coherence length of the photons emitted within the short space available between the source and the eye. Indeed, we were interested in imagining that the entire wavelength of the photon reached the eye. The coherence length of a typical NICHIA LED is of micrometer order, with a coherence time, $\tau_{coh}=\frac{L_{coh}}{c}$ (where $L_{coh}$is the coherence length (see Appendix 2) and *c* is the speed of light), of picosecond order. An LED wavelength of 520 nm was chosen, because eye functionality at maximum sensitivity under dark conditions requires activation of the rods that have the highest sensitivity to a wavelength of approximately 500−520 nm [1]. The classic slow-phase adaptation curve shows the change in sensitivity as a function of time [2,3] (S1 Fig).

However, a recent work [4] notes a number of biases in previous measurements and illustrates the methodological correctness of the most recent theoretical and empirical measures. The result is the presence of multiple peaks of maximum sensitivity for scotopic vision, along with a general increase in the range of wavelengths with several peak values, having a maximum at approximately 540 nm (S2 Fig).

These findings led us to choose a filter to further reduce the number of photons incident on the eye, and aided us in selecting a suitable frequency for the experiment. Ultimately, we chose the OptoSigma filter mentioned in the Materials and Methods section.

**Photomultiplier Characteristics and Measurements**

The photomultiplier used in the experiment was a Hamamatsu Photonics (Hamamatsu, Japan) [5] R 212 device, provided with an Amptek, Inc. (Bedford, MA) A-111 preamplifier-discriminator and a programmable counter PC05 TESYS (Trezzano, Italy) [6,7]. A Tektronix, Inc. (Beaverton, OR) 2245A 100MHz oscilloscope [8], was also used during the experiment. The photomultiplier was set according to the manufacturer's specifications to a bias scheme of 760 V, and was tuned to the minimum LED intensity, which was established as 61.8 μA, as explained below. Taking into account the *Q_e_* of 4% reported by the manufacturer, the average number of photons/s after a series of measurements appeared to be 433.33 (99% confidence interval [387.32, 499.33]. The dark count was 7.5 shots/s, which was a properly limited value.

**S1 Fig. Classic slow-phase adaptation curves.** After approximately 20-min exposure to dark conditions, the rods achieve maximum sensitivity.

**S2 Fig. Maximum sensitivity for scotopic vision (from [4], p. 168).** Overlay of measured data (solid line with experimental values) with theoretical prediction (dotted line).

References

1. Burle Electron Tubes. Electro-optics Handbook. Burle Electron Tubes; 1974.
2. Graham CH. Vision and visual perception. New York: John Wiley and Sons, Inc.; 1965.
3. Hecht S, Schlaer S, Pirenne MH. Energy, quanta and vision. J Opt Soc Am 1942;38: 196-208.
4. Fulton JT. Processes in biological vision. Vision Concepts. 2014. Available: [http://neuronresearch.net/vision/pdf/17Performance1a.pdf#page168](http://neuronresearch.net/vision/pdf/17Performance1a.pdf#page168).
5. Hamamatsu Co. Photomultiplier Tubes. Available: [http://www.hamamatsu.com](http://www.hamamatsu.com/).
6. Amptek Co. Charge sensitive preamplifiers. Available: http://www.amptek.com/hybrid-selection-guide/.
7. Tesys Technology & Systems SAS. Programmable Counters. Available: http://www.tesyslab.eu/en/8-programmable-counters-counting-systems**.**
8. Tektronix Inc. Oscilloscopes. Available: http://www.tek.com/oscilloscope#all.
