# Supplementary material for: Human Visual System as a Double-Slit Single Photon Interference Sensor: A Comparison between Modellistic and Biophysical Tests
Source: PLoS One. 2016 Jan 27;11(1):e0147464. doi: 10.1371/journal.pone.0147464 (PMC4729532; doi:10.1371/journal.pone.0147464)
Supplement: S2 Appendix — (DOC) [file pone.0147464.s002.doc]

**S2 Appendix**

**Bunching Problem**

A Poisson process with a rate of $\lambda$ is a counting process, $\left\{N(t)|t\geq0\right\}$, that possesses, among other characteristics, the property that the number of event occurrences counted within disjoint intervals are independent of each other. In fact, photons are completely uncorrelated (apart from coherent light) and temporal coherence indicates a monochromatic source. This is determined from the spectral width, $\Delta\nu$, where

$\tau_{coh}\cong\frac{1}{\Delta\nu}$ . (10)
This is because $L_{coh}$, during which light oscillates at the point of irradiation, has a regular and strongly periodic character. Here, $\Delta\nu$ is the spectral width (full width at half maximum) of the beam (in Hz). As light propagates at a rate of *c* $=3\times10^{11}$mm/s, the light oscillations are matched by the phase (i.e., they are coherent) over the length of the light propagation, $L_{coh}$(the measure of temporal or longitudinal coherence), such that

$L_{coh}=c\tau_{coh}=\frac{c}{\Delta\nu}$. (11)

The more monochromatic the light, the longer the length for which the light field is coherent in volume. For a single-mode (single-frequency) He-Ne laser ($\lambda$ = 632.8 nm), $L_{coh}$ >> 1 m.

As we did not use a laser source in this study, but instead applied an LED source that can be considered to be halfway between a perfect laser source and a thermal light [1-3], we must consider the possibility that photon bunching occurred [4,5]. Further, the photons may have followed a Bose-Einstein distribution (instead of a Poisson distribution), where

$P(n)=\frac{<n>^{n}}{(1+<n>)^{1+n}}$ . (12)

Here, if $<n>$ is the average number of photons per pulse, then

$<n>=\sum_{n=0}^{\infty} nP(n)$ . (13)

It must be noted that photon bunching can only be observed if the sampling time is much shorter than $\tau_{coh}$ [6]. This is because the light can no longer be considered to be monochromatic, if the observation time interval is comparable to or greater than $\tau_{coh}$. From the semi-classical perspective, the light intensity fluctuates randomly with the typical duration of the fluctuations, of the same order as $\tau_{coh}$. If a photon is detected within a small time interval, $\Delta t$, inside the field sampling time, $T_{\mathrm{int}}$, it is more likely that another photon will be detected within $\Delta t$. However, if $T_{\mathrm{int}}$ is greater than $\tau_{coh}$, a single mode will not be observed and the Bose-Einstein distribution has no application. The photon-count distribution then becomes the Poisson distribution. To avoid doubts that a Bose-Einstein distribution could emerge in our experiment, we wished to evaluate the $\tau_{coh}$ of our light source. For an LED emitting at $\lambda$ = 510 nm ($2\times10^{4}$ cm**^-1^**), for example, $\Delta\lambda$ = 37.3 nm ($2.7\times10^{5}$cm^-1^). Green LEDs (in particular NICHIA LEDs) are reported to have $L_{coh}$ ≅ 4 μm and $\Delta\lambda\cong30$ nm [7,8]. The $\tau_{coh}$ of our NICHIA LED was, therefore, of picosecond order. As $T_{\mathrm{int}}$ for the human eye can be set to the order of milliseconds, this means that Bose-Einstein distribution conditions were not applicable.

On the other hand, [9] shows that faint non-Poissonian sources with ultra-low mean photon numbers can be treated as being approximately Poissonian. In any case, in this study, a bunching condition was not an impediment to obtaining a possible experimental outcome, as we aimed to simulate the perception of interruptions in the photon stream. Such events are not prevented by the presence of photon bunches in the stream.

References

1. Woodward E. Photon counting statistics for two characteristic light sources. PhD Thesis, Rochester Institute of Technology. 2008.
2. Martinez Ricci ML, Mazzaferri J, Bragas AV, Martinez OE. Photon counting statistics using a digital oscilloscope. Am J Phys 2007;75: 707.
3. Mehta S, Saxena K, Dubey SK, Shakher C. Coherence characteristics of light-emitting diodes. JOL 2010;130(1): 96-102.
4. Hanbury Brown R, Twiss RQ. Interferometry of the intensity fluctuation in light. I. Basic Theory: the correlation between photons in coherent beams of radiation. Proc R Soc A 1957;242(1230): 300-324.
5. Purcell E. The question of correlation between photons in coherent light rays. Nature 1956;178(4541): 1046-1048.
6. Fry TC. Probability and its engineering uses. New York: Van Nostrand. 1929.
7. Kocayk P, Wiewior P, Radzewicz C. Photon counting statistics. Am J Phys 1996;64(3): 240-245.
8. Stuerwald S, Kemper B, Remmersmann C, Langehanenberg P, von Bally G. Application of light emitting diodes in digital holographic microscopy. Proc of SPIE 2008;6995: 699507-2008.
9. Hu Y, Peng X, Li T, Guo H. On the Poisson approximation to photon distribution for faint lasers. Phys Lett A 2007;367: 173-176.
