# Supplementary figures and images for: Human Visual System as a Double-Slit Single Photon Interference Sensor: A Comparison between Modellistic and Biophysical Tests
Source: PLoS One. 2016 Jan 27;11(1):e0147464. doi: 10.1371/journal.pone.0147464 (PMC4729532; doi:10.1371/journal.pone.0147464)

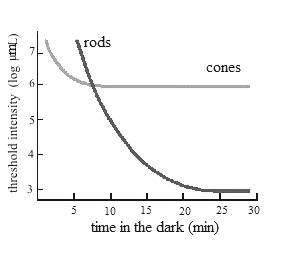

Supplement: S1 Fig — After approximately 20-min exposure to dark conditions, the rods achieve maximum sensitivity. (TIF) [file pone.0147464.s003.tif]

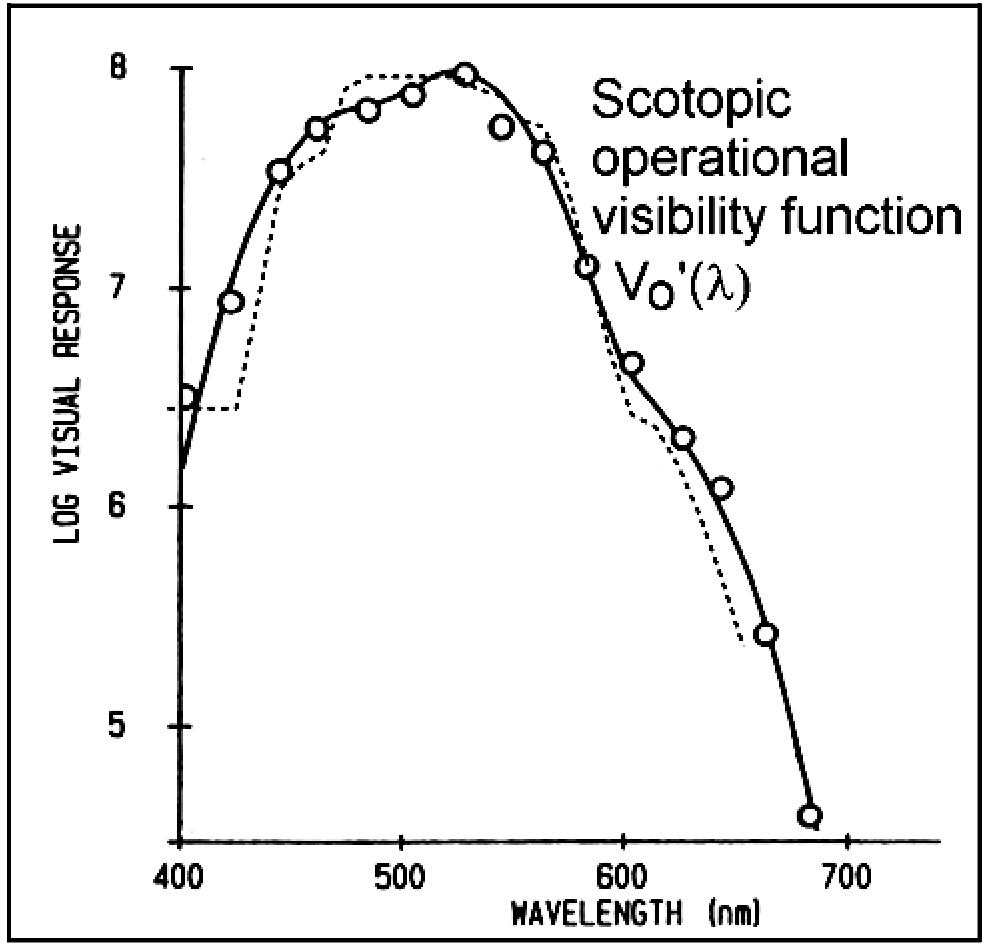

Supplement: S2 Fig — Overlay of measured data (solid line with experimental values) with theoretical prediction (dotted line). (TIF) [file pone.0147464.s004.tif]
